# National Health Systems and COVID-19 Death Toll Doubling Time

**DOI:** 10.3389/fpubh.2021.669038

**Published:** 2021-07-15

**Authors:** Miguel Alfaro, Diego Muñoz-Godoy, Manuel Vargas, Guillermo Fuertes, Claudia Duran, Rodrigo Ternero, Jorge Sabattin, Sebastian Gutierrez, Natalia Karstegl

**Affiliations:** ^1^Departamento de Ingeniería Industrial, Universidad de Santiago de Chile, Santiago, Chile; ^2^Facultad de Ingeniería y Tecnología, Universidad San Sebastián, Santiago, Chile; ^3^Facultad de Ingeniería, Ciencia y Tecnología, Universidad Bernardo O'Higgins, Santiago, Chile; ^4^Departamento de Industria, Facultad de Ingeniería, Universidad Tecnológica Metropolitana, Santiago, Chile; ^5^Escuela de Construcción, Universidad de las Américas, Santiago, Chile; ^6^Facultad de Ingeniería, Universidad Andres Bello, Santiago, Chile; ^7^Facultad de Economía, Gobierno y Comunicaciones, Universidad Central de Chile, Santiago, Chile; ^8^Facultad de Ciencias, Universidad Mayor, Chile, Santiago, Chile

**Keywords:** COVID-19, coronavirus, national health systems, death rate, non-parametric test

## Abstract

Coronavirus disease 2019 (COVID-19) has placed stress on all National Health Systems (NHSs) worldwide. Recent studies on the disease have evaluated different variables, namely, quarantine models, mitigation efforts, damage to mental health, mortality of the population with chronic diseases, diagnosis, use of masks and social distancing, and mortality based on age. This study focused on the four NHSs recognized by the WHO. These systems are as follows: (1) The Beveridge model, (2) the Bismarck model, (3) the National Health Insurance (NHI) model, and (4) the “Out-of-Pocket” model. The study analyzes the response of the health systems to the pandemic by comparing the time in days required to double the number of disease-related deaths. The statistical analysis was limited to 56 countries representing 70% of the global population. Each country was grouped into the health system defined by the WHO. The study compared the median death toll DT, between health systems using Mood's median test method. The results show high variability of the temporal trends in each group; none of the health systems for the three analyzed periods maintain stable interquartile ranges (IQRs). Nevertheless, the results obtained show similar medians between the study groups. The COVID-19 pandemic saturates health systems regardless of their management structures, and the result measured with the time for doubling death rate variable is similar among the four NHSs.

## Introduction

Over the last 15 months, coronavirus disease 2019 (COVID-19) has spread from China to the rest of the world. National Health Systems (NHSs) have implemented different strategies to control the disease (1–9) and to reduce the rate of infection and mortality. Different authorities have identified different factors associated with the distinctive dynamic system of the disease, such as, climatic factors (10–14), preexisting disease conditions (15), mental illnesses (16–21), prediction models (22) and (23), social distancing (24), and use of masks (25–28). Currently, the pandemic has more than 130 million confirmed cases worldwide with more than 2,800,000 deaths. America is the continent with the most active cases.

The capacity of NHSs is usually measured by health expenditure, health expenditure *per capita* (29, 30), and country classifications by income level or World Economic Situation and Prospects (WESP) classifier of the United Nations (31). Comparisons between the different health systems have been made by analyzing different quantitative and qualitative variables. The results allow the study of the performance of NHSs by comparing groups with different health requirements; each national health system creates action plans according to the most recurrent morbidities in its population according to its culture and lifestyle. The COVID pandemic allows us, for the first time, to compare different NHSs under the same global health requirement. The study evaluated the relationship between NHSs and COVID-19 death toll doubling time (DT) to evaluate the performance of different health systems in their response to the pandemic.

There are four types of NHSs. (1) The Beveridge model, which is financed by direct taxes, where the person being treated does not pay for care. In this model, most of the clinics and hospitals are owned by the government, and most of the employees are government employees. This system is implemented in countries such as Great Britain and Spain. (2) The Bismarck model is financed by private health insurance. Employers and employees jointly finance this system through payroll deduction. Medical care is paid for and reimbursed by insurance and health centers, and doctors are generally private. This system is applied in Germany, France, and Japan. (3) The NHI model is financed through payroll and tax deductions. These payments are turned over to the NHI program, which is administered by the government. Private hospitals and doctors provide health services. Some countries only allow the provision of health services to non-profit private foundations to achieve cost containment. This system is applied in countries like Canada and South Korea, among others. The last health system corresponds to the so-called (4) “Out-of-Pocket” system based on the lack of universal health coverage. In this system, the patient must pay for their health expenses. Those with the highest income will take care of themselves, and the poor will continue to be ill or die. This system is applied in Latin America, Africa, Eastern Europe, and Asia (32–34). Each system has its characteristics and different approaches, and they are heterogeneously distributed in different countries.

The study evaluated the relationship between NHSs and COVID-19 death toll DT to evaluate the performance of different health systems in their response to the pandemic.

## Materials and Methods

### The Four National Health Systems

The Beveridge model was founded in 1948 by William Beveridge. In this system, health coverage is universal; the healthcare system (HCS) is available to all citizens without direct payment, and the government finances it through taxes. The government owns most hospitals and clinics, and the officials are public employees. The state pays the healthcare, managing to regulate prices and to define the quality and costs of healthcare. Private clinics are also financed by the state, which defines the healthcare and the value of each health benefit. The greatest disadvantage of the system is the long waiting times for medical attention. This system is known globally as the National Health Service (NHS).

The Bismarck model was established by the Prussian Chancellor Otto von Bismarck during the German unification in the nineteenth century; this system uses an insurance system called sickness funds. These insurances are jointly financed between employers and employees under payroll discounts. In this case, all members contribute to a defined insurance fund, and this provides defined benefits. Private insurance companies must be non-profit; they exist for self-employed citizens and those who wish to receive elective services that sickness funds do not cover. This model has a multi-payer system, where each country has a different number of insurers to choose from. This system has universal coverage and stringent regulations with the sole objective of reaching the entire population and being a non-profit entity.

The NHI model is based on a universal and unique health insurance system defined in a geographic area and financed by the government through different sources such as taxes and social security contributions. The NHI model maintains strict independence between the universal insurance system provided by the government and the medical providers made up by foundations or private companies. A general benefit contract regulates the benefits. This contract allows (1) to limit an increase in providers, (2) to share the risk of diseases among the entire population, (3) to ensure the universal availability for all citizens, (4) to provide a general regulation for all providers, and (5) to ensure adequate and timely provision of medical services. The fundamental difference between the NHS and the NHI is the separation between universal government insurance and the provision of medical services. In the NHI system, the government negotiates prices and benefits with private companies and foundations; the government delegates the provision of all medical services to independent entities. This HCS has been adopted by the governments of Canada, Taiwan, and South Korea. Finally, the model based on universality does not require advertising expenses, reduces the cost of uncertainty by sharing risk, has no financial incentives for denial of service, does not cause bankruptcies in the population, and generates savings for the population due to its public nature.

The Out-of-Pocket model corresponds to health systems in impoverished and disorganized nations where the provision of medical services is not universal and corresponds to an economic transaction between the citizen and private medical service. Large areas of China, India, and rural Africa do not have adequate medical services for their population. About 40 industrialized countries maintain robust NHSs. The United States has a health system where each citizen can be classified in one of the four NHSs based on their social and economic position.

## Methodology

We reviewed the literature study regarding the mortality associated with the COVID-19 disease in different countries; however, the analysis was limited to 56 nations representing 70% of the global population. The countries were classified into one of the four NHS models. The information for classification was obtained from the WHO and the reports associated with each country. The characteristics of these nations are described in Table 1 with a mortality analysis of up to 360 days after the first 10 deaths in each country.

**Table 1:** DT of the number of deaths by country.

**Country**	**Population at 2020 estimated (K)**	**Period start**	**Total death as of the day: D0**	**Total death as of the day: D1** **(90 d)**	**Total death as of the day: D1** **(180 d)**	**Total death as of the day: D1** **(360 d)**	**Growth ratio: R** **(90 d)**	**Growth ratio: R** **(180 d)**	**Growth ratio: R** **(360 d)**	**Doubling time (day) (90 d)**	**Doubling time (day)** **(180 d)**	**Doubling time (day)** **(360 d)**	**Healthcare system**
USA	331,003	04-mar	11	109,589	183,801	513,849	9,962.6	16,709.2	46,713.5	**6.8**	**12.8**	**23.2**	Out-of-Pocket
Brazil	212,559	20-mar	11	47,748	134,106	279,286	4,340.7	12,191.5	25,389.6	**7.4**	**13.3**	**24.6**	Bismarck
Mexico	128,933	27-mar	12	25,06	74,949	198,239	2,088.3	6,245.8	16,519.9	**8.2**	**14.3**	**25.7**	Out-of-Pocket
UK	67,886	13-mar	10	39,186	41,683	124,801	3,918.6	4,168.3	12,480.1	**7.5**	**15.0**	**26.5**	Beveridge
India	1,380,004	23-mar	13	13,699	86,752	159,37	1,053.8	6,673.2	12,259.2	**9.0**	**14.2**	**26.5**	Out-of-Pocket
Russia	145,934	31-mar	10	9,152	20,239	95,41	915.2	2,023.9	9,541.0	**9.1**	**16.4**	**27.2**	Beveridge
Italy	60,462	25-feb	11	32,877	35,437	95,235	2,988.8	3,221.5	8,657.7	**7.8**	**15.4**	**27.5**	Beveridge
France	65,274	07-mar	10	29,114	30,717	87,373	2,911.4	3,071.7	8,737.3	**7.8**	**15.5**	**27.5**	Bismarck
Germany	83,784	15-mar	12	8,793	9,348	72,858	732.8	779.0	6,071.5	**9.5**	**18.7**	**28.6**	Bismarck
Colombia	50,883	29-mar	10	2,939	25,103	62,394	293.9	2,510.3	6,239.4	**11.0**	**15.9**	**28.6**	Bismarck
Iran	83,993	24-feb	12	7,417	20,502	59,264	618.1	1,708.5	4,938.7	**9.7**	**16.8**	**29.3**	Bismarck
South Africa	59,309	5-Apr	11	3,026	16,909	52,846	275.1	1,537.2	4,804.2	**11.1**	**17.0**	**29.4**	Beveridge
Poland	37,847	24-mar	10	1,359	2,293	48,807	135.9	229.3	4,880.7	**12.7**	**23.0**	**29.4**	Bismarck
Peru	32,972	27-mar	11	8,761	31,568	50,198	796.5	2,869.8	4,563,5	**9.3**	**15.7**	**29.6**	Out-of-Pocket
Argentina	45,196	27-mar	12	1,15	14,376	54,671	95.8	1,198.0	4,555.9	**13.7**	**17.6**	**29.6**	Bismarck
Ukraine	43,734	29-mar	11	1,121	3,91	32,368	101.9	355.5	2,942.5	**13.5**	**21.2**	**31.2**	Out-of-Pocket
Spain	46,755	07-mar	28	27,134	29,234	69,801	969.1	1,044.1	2,492.9	**9.1**	**17.9**	**31.9**	Beveridge
Canada	37,742	18-mar	10	8,521	9,249	22,426	852.1	924.9	2,242.6	**9.2**	**18.3**	**32.3**	National Health
Czechia	10,709	28-mar	11	349	567	25,055	31.7	51.5	2,277.7	**18.0**	**31.6**	**32.3**	Bismarck
Romania	19,238	24-mar	11	1,523	4,435	22,02	138.5	403.2	2,001.8	**12.7**	**20.8**	**32.8**	Bismarck
Indonesia	273,524	18-mar	19	2,231	8,841	38,329	117.4	465.3	2,017.3	**13.1**	**20.3**	**32.8**	Out-of-Pocket
Chile	19,116	31-mar	12	5,575	12,641	22,587	464.6	1,053.4	1,882.3	**10.2**	**17.9**	**33.1**	Bismarck
Hungary	9,66	25-mar	10	573	686	18,068	57.3	68.6	1,806.8	**15.4**	**29.5**	**33.3**	Bismarck
Belgium	11,59	17-mar	14	9,661	9,925	22,397	690.1	708.9	1,599.8	**9.5**	**19.0**	**33.8**	Bismarck
Turkey	84,339	22-mar	21	4,927	7,377	29,696	234.6	351.3	1,414.1	**11.4**	**21.3**	**34.4**	Beveridge
Portugal	10,197	21-mar	12	1,527	1,888	16,707	127.3	157.3	1,392.3	**12.9**	**24.7**	**34.5**	Beveridge
Netherlands	17,135	13-mar	12	6,063	6,281	15,99	505.3	523.4	1,332.5	**10.0**	**19.9**	**34.7**	Bismarck
Sweden	10,099	17-mar	10	4,891	5,846	13,146	489.1	584.6	1,314.6	**10.1**	**19.6**	**34.7**	Beveridge
Pakistan	220,892	26-mar	11	3,903	6,432	13,863	354.8	584.7	1,260.3	**10.6**	**19.6**	**35.0**	Out-of-Pocket
Iraq	40,223	14-mar	11	496	7,814	13,618	45.1	710.4	1,238.0	**16.4**	**19.0**	**35.0**	Out-of-Pocket
Ecuador	17,643	22-mar	14	4,156	11,044	16,3	296.9	788.9	1,164.3	**11.0**	**18.7**	**35.3**	Bismarck
Egypt	102,334	21-mar	10	2,017	5,715	11,384	201.7	571.5	1,138.4	**11.8**	**19.7**	**35.5**	Out-of-Pocket
Philippines	109,581	15-mar	12	1,074	4,108	12,545	89.5	342.3	1,045.4	**13.9**	**21.4**	**35.9**	Out-of-Pocket
Switzerland	8,655	13-mar	11	1,937	2,019	10,056	176.1	183.5	914.2	**12.1**	**23.9**	**36.6**	Bismarck
Morocco	36,911	26-mar	10	216	1,889	8,767	21.6	188.9	876.7	**20.3**	**23.8**	**36.8**	Out-of-Pocket
Bangladesh	164,689	6-Apr	12	2,052	5,325	9,105	171.0	443.8	758.8	**12.1**	**20.5**	**37.6**	Out-of-Pocket
Japan	126,476	09-mar	12	917	1,361	8,135	76.4	113.4	677.9	**14.4**	**26.4**	**38.3**	Bismarck
Saudi Arabia	34,814	31-mar	10	1,599	4,683	6,637	159.9	468.3	663.7	**12.3**	**20.3**	**38.4**	Beveridge
Israel	8,656	26-mar	10	314	1,36	6,092	31.4	136.0	609.2	**18.1**	**25.4**	**38.9**	Bismarck
Austria	9,006	22-mar	16	688	763	8,956	43.0	47.7	559.8	**16.6**	**32.3**	**39.4**	Bismarck
Greece	10,423	21-mar	13	189	325	7,196	14.5	25.0	553.5	**23.3**	**38.8**	**39.5**	Bismarck
Serbia	8,737	28-mar	10	265	745	5,002	26.5	74.5	500.2	**19.0**	**28.9**	**40.2**	Out-of-Pocket
Panama	4,315	28-mar	14	575	2,297	6,06	41.1	164.1	432.9	**16.8**	**24.5**	**41.1**	Out-of-Pocket
Moldova	4,034	4-Apr	12	572	1,336	4,915	47.7	111.3	409.6	**16.1**	**26.5**	**41.5**	Out-of-Pocket
N. Macedonia	2,083	1-Apr	10	302	729	3,642	30.2	72.9	364.2	**18.3**	**29.1**	**42.3**	Out-of-Pocket
Slovenia	2,079	29-mar	11	109	145	3,994	9.9	13.2	363.1	**27.2**	**48.4**	**42.3**	Bismarck
Dominican Republic	10,848	25-mar	10	675	2,054	3,269	67.5	205.4	326.9	**14.8**	**23.4**	**43.1**	Out-of-Pocket
Algeria	43,851	20-mar	10	811	1,645	3,04	81.1	164.5	304.0	**14.2**	**24.5**	**43.6**	Out-of-Pocket
China	1,439,324	22-Jan	17	4,636	4,646	4,797	272.7	273.3	282.2	**11.1**	**22.2**	**44.2**	Out-of-Pocket
Ireland	4,938	26-mar	19	1,726	1,792	4,587	90.8	94.3	241.4	**13.8**	**27.4**	**45.5**	Beveridge
Denmark	5,792	21-mar	13	600	635	2,396	46.2	48.8	184.3	**16.3**	**32.1**	**47.8**	Beveridge
S. Korea	51,269	25-feb	11	269	309	1,553	24.5	28.1	141.2	**19.5**	**37.4**	**50.4**	National Health
Malaysia	32,366	22-mar	10	121	129	1,22	12.1	12.9	122.0	**25.0**	**48.8**	**51.9**	Bismarck
Australia	25,5	26-mar	11	104	859	909	9.5	78.1	82.6	**27.8**	**28.6**	**56.5**	National Health
Finland	5,541	29-mar	11	328	343	809	29.8	31.2	73.5	**18.4**	**36.3**	**58.1**	Beveridge
Norway	5,421	23-mar	10	244	267	648	24.4	26.7	64.8	**19.5**	**38.0**	**59.8**	Beveridge

*Bold values represents comparative results (90, 180 and 360 days) for country taking into account DT death toll, between health systems*.

The death toll DT variable is presented in Equation 1. It represents the time in days it takes for the disease to double the number of deaths.

(1) $$\begin{array}{c} Dt=\frac{\text{Period Lenght}\;\;}{Log^{2}(\frac{D1}{D0})} \end{array}$$

where *D0* is the first day with accumulated deaths equal to 10 deaths, and *D1* is the day i with n accumulated deaths. The period length (*L*) corresponds to the time elapsed from the start day (baseline) to the evaluation day. The start day for each country was the date when 10 deaths were accumulated. Three study periods of 90, 180, and 360 days were considered for the study.

Non-parametric tests are used when the data do not meet the assumptions of normality and homoscedasticity. Assuming normality using goodness-of-fit tests must also consider the sample size of data. Statistical tests not adjusted to the sample size resulted in errors of the real distribution and established erroneous conclusions. Normality tests can be performed using the Kolmogorov–Smirnov, Kolmogorov–Smirnov–Lilliefors, Shapiro–Wilk, Anderson–Darling, and Jarque–Bera goodness-of-fit tests. For small sample sizes, the tests with the best non-normality detection power are Kolmogorov–Smirnov–Lilliefors and Anderson–Darling.

**Figure 1:**
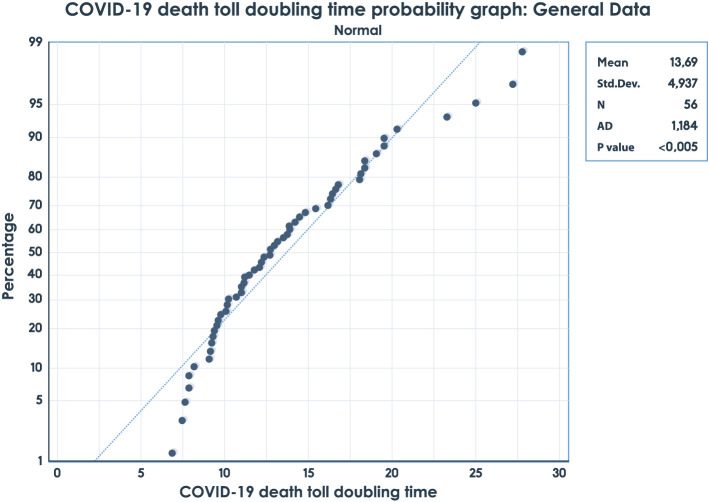
Anderson–Darling goodness-of-fit test for Bismarck Health System data.

We used the Anderson–Darling and Kolmogorov–Smirnov–Lilliefors goodness-of-fit tests for the samples of the COVID-19 death toll DT variable for each health system present in all the countries. The results of the normality goodness-of-fit tests are rejected for the studied samples; therefore, for the analysis of the samples of the COVID-19 death toll DT variable, non-parametric tests were used. The graphs represent the results of the Anderson–Darling goodness-of-fit test for the Bismarck Health System data (Figure 1) and the Anderson–Darling goodness-of-normality test for the general data of countries (Supplementary Figure 1).

The statistical model uses Mood's median test; in this study, we have one categorical factor and a continuous response of the distributions. Using this test, we can determine whether the medians of two or more groups differ. The selected level of significance is set at 0.05. The entire test was performed using the software MatLab.

The research study that proposes the statistical analysis of the comparison of medians of the death toll DT was performed with Mood's technique, using different cutoff dates to see the impact of the pandemic on each health system.

## Results

**Figure 2:**
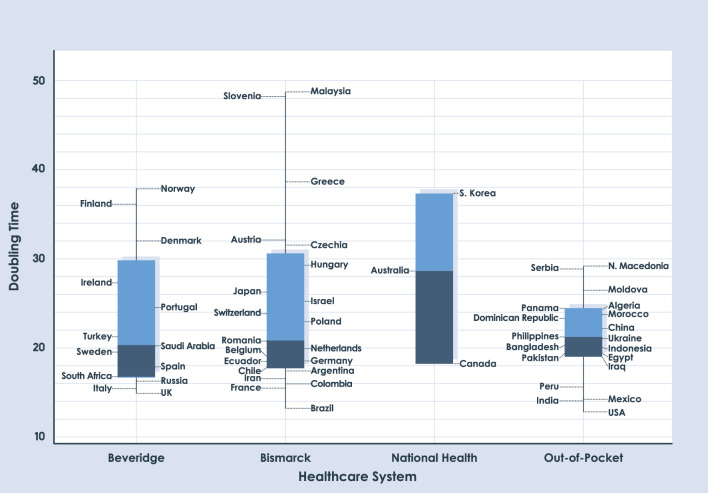
Box plot for DT of deaths with a period length of 180 days.

**Figure 3:**
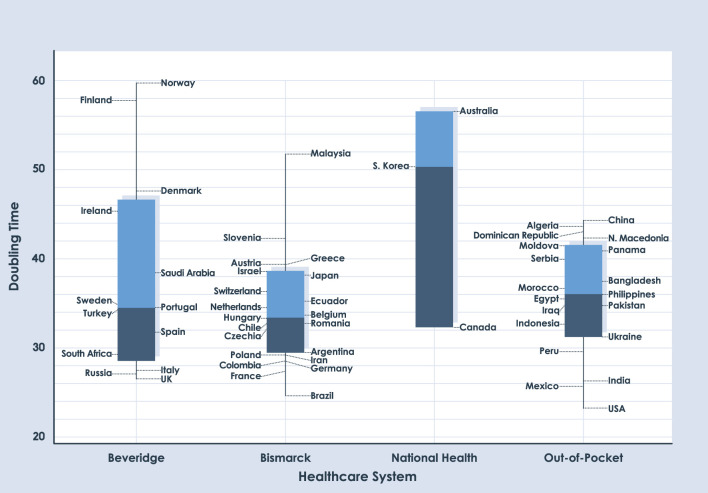
Box plot for DT of deaths with a period length of 360 days.

The analysis dates correspond to 90, 180, and 360 days after day 1, with a cumulative number of 10 deaths. For comparative analysis, this study only shows results of 180 and 360 days, the results of 90 days are shown in Supplementary Figure 2. Box charts in Figures 2, 3 show similar medians for each NHS. The two graphs demonstrate the difficulty of establishing COVID-19 trends. When comparing the three time periods which were analyzed, none of the health systems have a clear trend; the IQRs are not stable. Another interesting feature observed in Figures 2, 3 is the absence of outliers. No country has values outside the range of box charts (1.5 IQR).

We used Mood's median test to determine whether there are significant differences in the information for each date of the study. Based on Mood's non-parametric test, the distributions of these four groups were not significantly different when using a significance value of 0.05 (*p* = 0.671 for 90 days, *p*=0.917 for 180 days, and *p*=0.187 for 360 days). As seen in Table 1, the *p*-value for each date shows the absence of statistical differences between different health systems regarding their capacity to respond to the COVID-19 pandemic. Supplementary Tables 1–3 show the variables analyzed in the study.

## Discussion

Health policymakers are concerned about the performance of their NHSs, and many countries have introduced reforms aimed at improving performance. Different authors have established the capacity of a health system according to (1) its structure, (2) the health expenditure concerning the gross domestic product (GDP), (3) some indicators of development of a country, (4) or a combination of the aforementioned variables.

Some research studies consider that increasing resources for NHSs are critical to improving health in developing countries. Still, in most countries, significant progress can be made by using existing resources more efficiently. The efficiency of NHSs depends directly on the organizational structure; this research study classifies countries according to their NHS and compares the results of these classifications regarding health performance in the face of the pandemic. This comparison is made under the variable COVID-19 death toll DT.

The four NHSs compared in this study were as follows: (1) The Beveridge model, (2) the Bismarck model, (3) the NHI model, and (4) the “Out-of-Pocket” model. The classification of each model in different countries has been studied by international organizations such as the WHO.

The tables show eight countries with the highest mortality doubling rates in all ranges (90, 180, and 360 days). Those countries are the USA, Brazil, the UK, Italy, France, Mexico, India, and Russia. In addition, the USA is the country in the study with the highest doubling death rate in the three time ranges.

There is no clear trend based on the health system; in each time period analyzed, the IQRs are not stable. The absence of outliers is observed in the graphs, which is an uncommon phenomenon in box plots. No country has values outside the range of box charts.

The *p*-values of the different time periods (180 and 360 days) show no significant difference in the COVID-19 death toll DT between the different NHSs. Mood's median test confirms with a *p*-value of 0.05 that the four NHSs do not have statistical differences of the median.

In the future, the use of this methodology will allow new analyses of NHSs to be carried out against other global health requirements, for example, vaccination against COVID-19, HIV/AIDS, and new emerging diseases.

We used MatLab software to develop this study. The databases of the three study periods of 90, 180, and 360 days and the function code to execute in MatLab are available in the GitHub repository.

## Conclusions

The COVID-19 pandemic has been studied from numerous aspects, namely, climatic factors, preexisting disease conditions, mental illnesses, quarantine models, social distancing, age, and use of masks, among others.

This study evaluated the relationship between the NHS and the COVID-19 death toll DT of each country to evaluate their performance during the pandemic.

The study establishes no difference in the performance of the different NHSs during the COVID-19 pandemic. However, multidimensional modeling must be carried out to discover the causes of COVID-19 death toll DT.

## Data Availability Statement

The datasets generated for this study can be found in online repositories. The names of the repository/repositories and accession number(s) can be found at: https://github.com/owid/covid-19-data/tree/master/public/data.

## Author Contributions

All authors listed have made a substantial, direct and intellectual contribution to the work, and approved it for publication.

## Conflict of Interest

The authors declare that the research was conducted in the absence of any commercial or financial relationships that could be construed as a potential conflict of interest.

## Supplementary Material

Supplementary Figure 1 Anderson-Darling goodness-of-fit test for general country data.

Supplementary Figure 2 Box Plot for doubling time of deaths with period length 90 days.
