# Supplementary material for: National Health Systems and COVID-19 Death Toll Doubling Time
Source: Front Public Health. 2021 Jul 15;9:669038. doi: 10.3389/fpubh.2021.669038 (PMC8319632; doi:10.3389/fpubh.2021.669038)
Supplement: Supplementary file 1 [file Table_1.DOCX]

Complementary Table 1. Doubling time of number of deaths by country with period length 90 days.

| Country | Healthcare system | Population at 2020 estimated (K)  min 1M | Period start  death >=10 | Total death as of the day: D0 | Period end | Total death as of the day: D1 | Period length L (day) | Growth ratio: R  =D1/D0 | Doubling time (day)  =L/log^2^(R) |
| --- | --- | --- | --- | --- | --- | --- | --- | --- | --- |
| USA | Out-of-Pocket | 331,003 | 4-Mar | 11 | 2-Jun | 109,589 | 90 | 9,962.6 | 6.8 |
| Brazil | Bismarck | 212,559 | 20-Mar | 11 | 18-Jun | 47,748 | 90 | 4,340.7 | 7.4 |
| UK | Beveridge | 67,886 | 13-Mar | 10 | 11-Jun | 39,186 | 90 | 3,918.6 | 7.5 |
| Italy | Beveridge | 60,462 | 25-Feb | 11 | 25-May | 32,877 | 90 | 2,988.8 | 7.8 |
| France | Bismarck | 65,274 | 7-Mar | 10 | 5-Jun | 29,114 | 90 | 2,911.4 | 7.8 |
| Mexico | Out-of-Pocket | 128,933 | 27-Mar | 12 | 25-Jun | 25,060 | 90 | 2,088.3 | 8.2 |
| India | Out-of-Pocket | 1,380,004 | 23-Mar | 13 | 21-Jun | 13,699 | 90 | 1,053.8 | 9.0 |
| Spain | Beveridge | 46,755 | 7-Mar | 28 | 5-Jun | 27,134 | 90 | 969.1 | 9.1 |
| Russia | Beveridge | 145,934 | 31-Mar | 10 | 29-Jun | 9,152 | 90 | 915.2 | 9.1 |
| Canada | National Health | 37,742 | 18-Mar | 10 | 16-Jun | 8,521 | 90 | 852.1 | 9.2 |
| Peru | Out-of-Pocket | 32,972 | 27-Mar | 11 | 25-jun | 8,761 | 90 | 796.5 | 9.3 |
| Germany | Bismarck | 83,784 | 15-Mar | 12 | 13-Jun | 8,793 | 90 | 732.8 | 9.5 |
| Belgium | Bismarck | 11,590 | 17-Mar | 14 | 15-Jun | 9,661 | 90 | 690.1 | 9.5 |
| Iran | Bismarck | 83,993 | 24-Feb | 12 | 24-May | 7,417 | 90 | 618.1 | 9.7 |
| Netherlands | Bismarck | 17,135 | 13-Mar | 12 | 11-Jun | 6,063 | 90 | 505.3 | 10.0 |
| Sweden | Beveridge | 10,099 | 17-Mar | 10 | 15-Jun | 4,891 | 90 | 489.1 | 10.1 |
| Chile | Bismarck | 19,116 | 31-Mar | 12 | 29-Jun | 5,575 | 90 | 464.6 | 10.2 |
| Pakistan | Out-of-Pocket | 220,892 | 26-Mar | 11 | 24-Jun | 3,903 | 90 | 354.8 | 10.6 |
| Ecuador | Bismarck | 17,643 | 22-Mar | 14 | 20-Jun | 4,156 | 90 | 296.9 | 11.0 |
| Colombia | Bismarck | 50,883 | 29-Mar | 10 | 27-Jun | 2,939 | 90 | 293.9 | 11.0 |
| South Africa | Beveridge | 59,309 | 5-Apr | 11 | 4-Jul | 3,026 | 90 | 275.1 | 11.1 |
| China | Out-of-Pocket | 1,439,324 | 22-Jan | 17 | 21-Apr | 4,636 | 90 | 272.7 | 11.1 |
| Turkey | Beveridge | 84,339 | 22-Mar | 21 | 20-Jun | 4,927 | 90 | 234.6 | 11.4 |
| Egypt | Out-of-Pocket | 102,334 | 21-Mar | 10 | 19-Jun | 2,017 | 90 | 201.7 | 11.8 |
| Switzerland | Bismarck | 8,655 | 13-Mar | 11 | 11-Jun | 1,937 | 90 | 176.1 | 12.1 |
| Bangladesh | Out-of-Pocket | 164,689 | 6-Apr | 12 | 5-Jul | 2,052 | 90 | 171.0 | 12.1 |
| Saudi Arabia | Beveridge | 34,814 | 31-Mar | 10 | 29-Jun | 1,599 | 90 | 159.9 | 12.3 |
| Romania | Bismarck | 19,238 | 24-Mar | 11 | 22-Jun | 1,523 | 90 | 138.5 | 12.7 |
| Poland | Bismarck | 37,847 | 24-Mar | 10 | 22-Jun | 1,359 | 90 | 135.9 | 12.7 |
| Portugal | Beveridge | 10,197 | 21-Mar | 12 | 19-Jun | 1,527 | 90 | 127.3 | 12.9 |
| Indonesia | Out-of-Pocket | 273,524 | 18-Mar | 19 | 16-Jun | 2,231 | 90 | 117.4 | 13.1 |
| Ukraine | Out-of-Pocket | 43,734 | 29-Mar | 11 | 27-Jun | 1,121 | 90 | 101.9 | 13.5 |
| Argentina | Bismarck | 45,196 | 27-Mar | 12 | 25-Jun | 1,150 | 90 | 95.8 | 13.7 |
| Ireland | Beveridge | 4,938 | 26-Mar | 19 | 24-Jun | 1,726 | 90 | 90.8 | 13.8 |
| Philippines | Out-of-Pocket | 109,581 | 15-Mar | 12 | 13-Jun | 1,074 | 90 | 89.5 | 13.9 |
| Algeria | Out-of-Pocket | 43,851 | 20-Mar | 10 | 18-Jun | 811 | 90 | 81.1 | 14.2 |
| Japan | Bismarck | 126,476 | 9-Mar | 12 | 7-Jun | 917 | 90 | 76.4 | 14.4 |
| Dominican Republic | Out-of-Pocket | 10,848 | 25-Mar | 10 | 23-Jun | 675 | 90 | 67.5 | 14.8 |
| Hungary | Bismarck | 9,660 | 25-Mar | 10 | 23-Jun | 573 | 90 | 57.3 | 15.4 |
| Moldova | Out-of-Pocket | 4,034 | 4-Apr | 12 | 3-Jul | 572 | 90 | 47.7 | 16.1 |
| Denmark | Beveridge | 5,792 | 21-Mar | 13 | 19-Jun | 600 | 90 | 46.2 | 16.3 |
| Iraq | Out-of-Pocket | 40,223 | 14-Mar | 11 | 12-Jun | 496 | 90 | 45.1 | 16.4 |
| Austria | Bismarck | 9,006 | 22-Mar | 16 | 20-Jun | 688 | 90 | 43.0 | 16.6 |
| Panama | Out-of-Pocket | 4,315 | 28-Mar | 14 | 26-jun | 575 | 90 | 41.1 | 16.8 |
| Czechia | Bismarck | 10,709 | 28-Mar | 11 | 26-jun | 349 | 90 | 31.7 | 18.0 |
| Israel | Bismarck | 8,656 | 26-Mar | 10 | 24-Jun | 314 | 90 | 31.4 | 18.1 |
| N. Macedonia | Out-of-Pocket | 2,083 | 1-Apr | 10 | 30-Jun | 302 | 90 | 30.2 | 18.3 |
| Finland | Beveridge | 5,541 | 29-Mar | 11 | 27-Jun | 328 | 90 | 29.8 | 18.4 |
| Serbia | Out-of-Pocket | 8,737 | 28-Mar | 10 | 26-Jun | 265 | 90 | 26.5 | 19.0 |
| S. Korea | National Health | 51,269 | 25-Feb | 11 | 25-May | 269 | 90 | 24.5 | 19.5 |
| Norway | Beveridge | 5,421 | 23-Mar | 10 | 21-Jun | 244 | 90 | 24.4 | 19.5 |
| Morocco | Out-of-Pocket | 36,911 | 26-Mar | 10 | 24-Jun | 216 | 90 | 21.6 | 20.3 |
| Greece | Bismarck | 10,423 | 21-Mar | 13 | 19-Jun | 189 | 90 | 14.5 | 23.3 |
| Malaysia | Bismarck | 32,366 | 22-Mar | 10 | 20-Jun | 121 | 90 | 12.1 | 25.0 |
| Slovenia | Bismarck | 2,079 | 29-Mar | 11 | 27-Jun | 109 | 90 | 9.9 | 27.2 |
| Australia | National Health | 25,500 | 26-Mar | 11 | 24-Jun | 104 | 90 | 9.5 | 27.8 |

Complementary Table 2. Doubling time of number of deaths by country with period length 180 days.

| Country | Healthcare system | Population at 2020 estimated (K)  min 1M | Period start  death >=10 | Total death as of the day: D0 | Period end | Total death as of the day: D1 | Period length L (day) | Growth ratio: R  =D1/D0 | Doubling time (day)  =L/log^2^(R) |
| --- | --- | --- | --- | --- | --- | --- | --- | --- | --- |
| USA | Out-of-Pocket | 331,003 | 4-Mar | 11 | 31-Aug | 183,801 | 180 | 16,709.2 | 12.8 |
| Brazil | Bismarck | 212,559 | 20-Mar | 11 | 16-Sep | 134,106 | 180 | 12,191.5 | 13.3 |
| India | Out-of-Pocket | 1,380,004 | 23-Mar | 13 | 19-Sep | 86,752 | 180 | 6,673.2 | 14.2 |
| Mexico | Out-of-Pocket | 128,933 | 27-Mar | 12 | 23-Sep | 74,949 | 180 | 6,245.8 | 14.3 |
| UK | Beveridge | 67,886 | 13-Mar | 10 | 9-Sep | 41,683 | 180 | 4,168.3 | 15.0 |
| Italy | Beveridge | 60,462 | 25-Feb | 11 | 23-Aug | 35,437 | 180 | 3,221.5 | 15.4 |
| France | Bismarck | 65,274 | 7-Mar | 10 | 3-Sep | 30,717 | 180 | 3,071.7 | 15.5 |
| Peru | Out-of-Pocket | 32,972 | 27-Mar | 11 | 23-sept | 31,568 | 180 | 2,869.8 | 15.7 |
| Colombia | Bismarck | 50,883 | 29-Mar | 10 | 25-Sep | 25,103 | 180 | 2,510.3 | 15.9 |
| Russia | Beveridge | 145,934 | 31-Mar | 10 | 27-Sep | 20,239 | 180 | 2,023.9 | 16.4 |
| Iran | Bismarck | 83,993 | 24-Feb | 12 | 22-Aug | 20,502 | 180 | 1,708.5 | 16.8 |
| South Africa | Beveridge | 59,309 | 5-Apr | 11 | 2-Oct | 16,909 | 180 | 1,537.2 | 17.0 |
| Argentina | Bismarck | 45,196 | 27-Mar | 12 | 23-Sep | 14,376 | 180 | 1,198.0 | 17.6 |
| Chile | Bismarck | 19,116 | 31-Mar | 12 | 27-Sep | 12,641 | 180 | 1,053.4 | 17.9 |
| Spain | Beveridge | 46,755 | 7-Mar | 28 | 3-Sep | 29,234 | 180 | 1,044.1 | 17.9 |
| Canada | National Health | 37,742 | 18-Mar | 10 | 14-Sep | 9,249 | 180 | 924.9 | 18.3 |
| Ecuador | Bismarck | 17,643 | 22-Mar | 14 | 18-Sep | 11,044 | 180 | 788.9 | 18.7 |
| Germany | Bismarck | 83,784 | 15-Mar | 12 | 11-Sep | 9,348 | 180 | 779.0 | 18.7 |
| Iraq | Out-of-Pocket | 40,223 | 14-Mar | 11 | 10-Sep | 7,814 | 180 | 710.4 | 19.0 |
| Belgium | Bismarck | 11,590 | 17-Mar | 14 | 13-Sep | 9,925 | 180 | 708.9 | 19.0 |
| Pakistan | Out-of-Pocket | 220,892 | 26-Mar | 11 | 22-Sep | 6,432 | 180 | 584.7 | 19.6 |
| Sweden | Beveridge | 10,099 | 17-Mar | 10 | 13-Sep | 5,846 | 180 | 584.6 | 19.6 |
| Egypt | Out-of-Pocket | 102,334 | 21-Mar | 10 | 17-Sep | 5,715 | 180 | 571.5 | 19.7 |
| Netherlands | Bismarck | 17,135 | 13-Mar | 12 | 9-Sep | 6,281 | 180 | 523.4 | 19.9 |
| Saudi Arabia | Beveridge | 34,814 | 31-Mar | 10 | 27-Sep | 4,683 | 180 | 468.3 | 20.3 |
| Indonesia | Out-of-Pocket | 273,524 | 18-Mar | 19 | 14-Sep | 8,841 | 180 | 465.3 | 20.3 |
| Bangladesh | Out-of-Pocket | 164,689 | 6-Apr | 12 | 3-Oct | 5,325 | 180 | 443.8 | 20.5 |
| Romania | Bismarck | 19,238 | 24-Mar | 11 | 20-Sep | 4,435 | 180 | 403.2 | 20.8 |
| Ukraine | Out-of-Pocket | 43,734 | 29-Mar | 11 | 25-Sep | 3,910 | 180 | 355.5 | 21.2 |
| Turkey | Beveridge | 84,339 | 22-Mar | 21 | 18-Sep | 7,377 | 180 | 351.3 | 21.3 |
| Philippines | Out-of-Pocket | 109,581 | 15-Mar | 12 | 11-Sep | 4,108 | 180 | 342.3 | 21.4 |
| China | Out-of-Pocket | 1,439,324 | 22-Jan | 17 | 20-Jul | 4,646 | 180 | 273.3 | 22.2 |
| Poland | Bismarck | 37,847 | 24-Mar | 10 | 20-Sep | 2,293 | 180 | 229.3 | 23.0 |
| Dominican Republic | Out-of-Pocket | 10,848 | 25-Mar | 10 | 21-Sep | 2,054 | 180 | 205.4 | 23.4 |
| Morocco | Out-of-Pocket | 36,911 | 26-Mar | 10 | 22-Sep | 1,889 | 180 | 188.9 | 23.8 |
| Switzerland | Bismarck | 8,655 | 13-Mar | 11 | 9-Sep | 2,019 | 180 | 183.5 | 23.9 |
| Algeria | Out-of-Pocket | 43,851 | 20-Mar | 10 | 16-Sep | 1,645 | 180 | 164.5 | 24.5 |
| Panama | Out-of-Pocket | 4,315 | 28-Mar | 14 | 24-sept | 2,297 | 180 | 164.1 | 24.5 |
| Portugal | Beveridge | 10,197 | 21-Mar | 12 | 17-Sep | 1,888 | 180 | 157.3 | 24.7 |
| Israel | Bismarck | 8,656 | 26-Mar | 10 | 22-Sep | 1,360 | 180 | 136.0 | 25.4 |
| Japan | Bismarck | 126,476 | 9-Mar | 12 | 5-Sep | 1,361 | 180 | 113.4 | 26.4 |
| Moldova | Out-of-Pocket | 4,034 | 4-Apr | 12 | 1-Oct | 1,336 | 180 | 111.3 | 26.5 |
| Ireland | Beveridge | 4,938 | 26-Mar | 19 | 22-Sep | 1,792 | 180 | 94.3 | 27.4 |
| Australia | National Health | 25,500 | 26-Mar | 11 | 22-Sep | 859 | 180 | 78.1 | 28.6 |
| Serbia | Out-of-Pocket | 8,737 | 28-Mar | 10 | 24-Sep | 745 | 180 | 74.5 | 28.9 |
| N. Macedonia | Out-of-Pocket | 2,083 | 1-Apr | 10 | 28-Sep | 729 | 180 | 72.9 | 29.1 |
| Hungary | Bismarck | 9,660 | 25-Mar | 10 | 21-Sep | 686 | 180 | 68.6 | 29.5 |
| Czechia | Bismarck | 10,709 | 28-Mar | 11 | 24-sept | 567 | 180 | 51.5 | 31.6 |
| Denmark | Beveridge | 5,792 | 21-Mar | 13 | 17-Sep | 635 | 180 | 48.8 | 32.1 |
| Austria | Bismarck | 9,006 | 22-Mar | 16 | 18-Sep | 763 | 180 | 47.7 | 32.3 |
| Finland | Beveridge | 5,541 | 29-Mar | 11 | 25-Sep | 343 | 180 | 31.2 | 36.3 |
| S. Korea | National Health | 51,269 | 25-Feb | 11 | 23-Aug | 309 | 180 | 28.1 | 37.4 |
| Norway | Beveridge | 5,421 | 23-Mar | 10 | 19-Sep | 267 | 180 | 26.7 | 38.0 |
| Greece | Bismarck | 10,423 | 21-Mar | 13 | 17-Sep | 325 | 180 | 25.0 | 38.8 |
| Slovenia | Bismarck | 2,079 | 29-Mar | 11 | 25-Sep | 145 | 180 | 13.2 | 48.4 |
| Malaysia | Bismarck | 32,366 | 22-Mar | 10 | 18-Sep | 129 | 180 | 12.9 | 48.8 |

Complementary Table 3. Doubling time of number of deaths by country with period length 360 days.

| Country | Healthcare system | Population at 2020 estimated (K)  min 1M | Period start  death >=10 | Total death as of the day: D0 | Period end | Total death as of the day: D1 | Period length L (day) | Growth ratio: R  =D1/D0 | Doubling time (day)  =L/log^2^(R) |
| --- | --- | --- | --- | --- | --- | --- | --- | --- | --- |
| USA | Out-of-Pocket | 331,003 | 4-Mar | 11 | 27-Feb | 513,849 | 360 | 46,713.5 | 23.2 |
| Brazil | Bismarck | 212,559 | 20-Mar | 11 | 15-Mar | 279,286 | 360 | 25,389.6 | 24.6 |
| Mexico | Out-of-Pocket | 128,933 | 27-Mar | 12 | 22-Mar | 198,239 | 360 | 16,519.9 | 25.7 |
| UK | Beveridge | 67,886 | 13-Mar | 10 | 8-Mar | 124,801 | 360 | 12,480.1 | 26.5 |
| India | Out-of-Pocket | 1,380,004 | 23-Mar | 13 | 18-Mar | 159,370 | 360 | 12,259.2 | 26.5 |
| Russia | Beveridge | 145,934 | 31-Mar | 10 | 26-Mar | 95,410 | 360 | 9,541.0 | 27.2 |
| France | Bismarck | 65,274 | 7-Mar | 10 | 2-Mar | 87,373 | 360 | 8,737.3 | 27.5 |
| Italy | Beveridge | 60,462 | 25-Feb | 11 | 19-Feb | 95,235 | 360 | 8,657.7 | 27.5 |
| Colombia | Bismarck | 50,883 | 29-Mar | 10 | 24-Mar | 62,394 | 360 | 6,239.4 | 28.6 |
| Germany | Bismarck | 83,784 | 15-Mar | 12 | 10-Mar | 72,858 | 360 | 6,071.5 | 28.6 |
| Iran | Bismarck | 83,993 | 24-Feb | 12 | 18-Feb | 59,264 | 360 | 4,938.7 | 29.3 |
| Poland | Bismarck | 37,847 | 24-Mar | 10 | 19-Mar | 48,807 | 360 | 4,880.7 | 29.4 |
| South Africa | Beveridge | 59,309 | 5-Apr | 11 | 31-Mar | 52,846 | 360 | 4,804.2 | 29.4 |
| Peru | Out-of-Pocket | 32,972 | 27-Mar | 11 | 22-mar | 50,198 | 360 | 4,563.5 | 29.6 |
| Argentina | Bismarck | 45,196 | 27-Mar | 12 | 22-Mar | 54,671 | 360 | 4,555.9 | 29.6 |
| Ukraine | Out-of-Pocket | 43,734 | 29-Mar | 11 | 24-Mar | 32,368 | 360 | 2,942.5 | 31.2 |
| Spain | Beveridge | 46,755 | 7-Mar | 28 | 2-Mar | 69,801 | 360 | 2,492.9 | 31.9 |
| Czechia | Bismarck | 10,709 | 28-Mar | 11 | 23-mar | 25,055 | 360 | 2,277.7 | 32.3 |
| Canada | National Health | 37,742 | 18-Mar | 10 | 13-Mar | 22,426 | 360 | 2,242.6 | 32.3 |
| Indonesia | Out-of-Pocket | 273,524 | 18-Mar | 19 | 13-Mar | 38,329 | 360 | 2,017.3 | 32.8 |
| Romania | Bismarck | 19,238 | 24-Mar | 11 | 19-Mar | 22,020 | 360 | 2,001.8 | 32.8 |
| Chile | Bismarck | 19,116 | 31-Mar | 12 | 26-Mar | 22,587 | 360 | 1,882.3 | 33.1 |
| Hungary | Bismarck | 9,660 | 25-Mar | 10 | 20-Mar | 18,068 | 360 | 1,806.8 | 33.3 |
| Belgium | Bismarck | 11,590 | 17-Mar | 14 | 12-Mar | 22,397 | 360 | 1,599.8 | 33.8 |
| Turkey | Beveridge | 84,339 | 22-Mar | 21 | 17-Mar | 29,696 | 360 | 1,414.1 | 34.4 |
| Portugal | Beveridge | 10,197 | 21-Mar | 12 | 16-Mar | 16,707 | 360 | 1,392.3 | 34.5 |
| Netherlands | Bismarck | 17,135 | 13-Mar | 12 | 8-Mar | 15,990 | 360 | 1,332.5 | 34.7 |
| Sweden | Beveridge | 10,099 | 17-Mar | 10 | 12-Mar | 13,146 | 360 | 1,314.6 | 34.7 |
| Pakistan | Out-of-Pocket | 220,892 | 26-Mar | 11 | 21-Mar | 13,863 | 360 | 1,260.3 | 35.0 |
| Iraq | Out-of-Pocket | 40,223 | 14-Mar | 11 | 9-Mar | 13,618 | 360 | 1,238.0 | 35.0 |
| Ecuador | Bismarck | 17,643 | 22-Mar | 14 | 17-Mar | 16,300 | 360 | 1,164.3 | 35.3 |
| Egypt | Out-of-Pocket | 102,334 | 21-Mar | 10 | 16-Mar | 11,384 | 360 | 1,138.4 | 35.5 |
| Philippines | Out-of-Pocket | 109,581 | 15-Mar | 12 | 10-Mar | 12,545 | 360 | 1,045.4 | 35.9 |
| Switzerland | Bismarck | 8,655 | 13-Mar | 11 | 8-Mar | 10,056 | 360 | 914.2 | 36.6 |
| Morocco | Out-of-Pocket | 36,911 | 26-Mar | 10 | 21-Mar | 8,767 | 360 | 876.7 | 36.8 |
| Bangladesh | Out-of-Pocket | 164,689 | 6-Apr | 12 | 1-Apr | 9,105 | 360 | 758.8 | 37.6 |
| Japan | Bismarck | 126,476 | 9-Mar | 12 | 4-Mar | 8,135 | 360 | 677.9 | 38.3 |
| Saudi Arabia | Beveridge | 34,814 | 31-Mar | 10 | 26-Mar | 6,637 | 360 | 663.7 | 38.4 |
| Israel | Bismarck | 8,656 | 26-Mar | 10 | 21-Mar | 6,092 | 360 | 609.2 | 38.9 |
| Austria | Bismarck | 9,006 | 22-Mar | 16 | 17-Mar | 8,956 | 360 | 559.8 | 39.4 |
| Greece | Bismarck | 10,423 | 21-Mar | 13 | 16-Mar | 7,196 | 360 | 553.5 | 39.5 |
| Serbia | Out-of-Pocket | 8,737 | 28-Mar | 10 | 23-Mar | 5,002 | 360 | 500.2 | 40.2 |
| Panama | Out-of-Pocket | 4,315 | 28-Mar | 14 | 23-mar | 6,060 | 360 | 432.9 | 41.1 |
| Moldova | Out-of-Pocket | 4,034 | 4-Apr | 12 | 30-Mar | 4,915 | 360 | 409.6 | 41.5 |
| N. Macedonia | Out-of-Pocket | 2,083 | 1-Apr | 10 | 27-Mar | 3,642 | 360 | 364.2 | 42.3 |
| Slovenia | Bismarck | 2,079 | 29-Mar | 11 | 24-Mar | 3,994 | 360 | 363.1 | 42.3 |
| Dominican Republic | Out-of-Pocket | 10,848 | 25-Mar | 10 | 20-Mar | 3,269 | 360 | 326.9 | 43.1 |
| Algeria | Out-of-Pocket | 43,851 | 20-Mar | 10 | 15-Mar | 3,040 | 360 | 304.0 | 43.6 |
| China | Out-of-Pocket | 1,439,324 | 22-Jan | 17 | 16-Jan | 4,797 | 360 | 282.2 | 44.2 |
| Ireland | Beveridge | 4,938 | 26-Mar | 19 | 21-Mar | 4,587 | 360 | 241.4 | 45.5 |
| Denmark | Beveridge | 5,792 | 21-Mar | 13 | 16-Mar | 2,396 | 360 | 184.3 | 47.8 |
| S. Korea | National Health | 51,269 | 25-Feb | 11 | 19-Feb | 1,553 | 360 | 141.2 | 50.4 |
| Malaysia | Bismarck | 32,366 | 22-Mar | 10 | 17-Mar | 1,220 | 360 | 122.0 | 51.9 |
| Australia | National Health | 25,500 | 26-Mar | 11 | 21-Mar | 909 | 360 | 82.6 | 56.5 |
| Finland | Beveridge | 5,541 | 29-Mar | 11 | 24-Mar | 809 | 360 | 73.5 | 58.1 |
| Norway | Beveridge | 5,421 | 23-Mar | 10 | 18-Mar | 648 | 360 | 64.8 | 59.8 |
