# Supplementary figures and images for: National Health Systems and COVID-19 Death Toll Doubling Time
Source: Front Public Health. 2021 Jul 15;9:669038. doi: 10.3389/fpubh.2021.669038 (PMC8319632; doi:10.3389/fpubh.2021.669038)

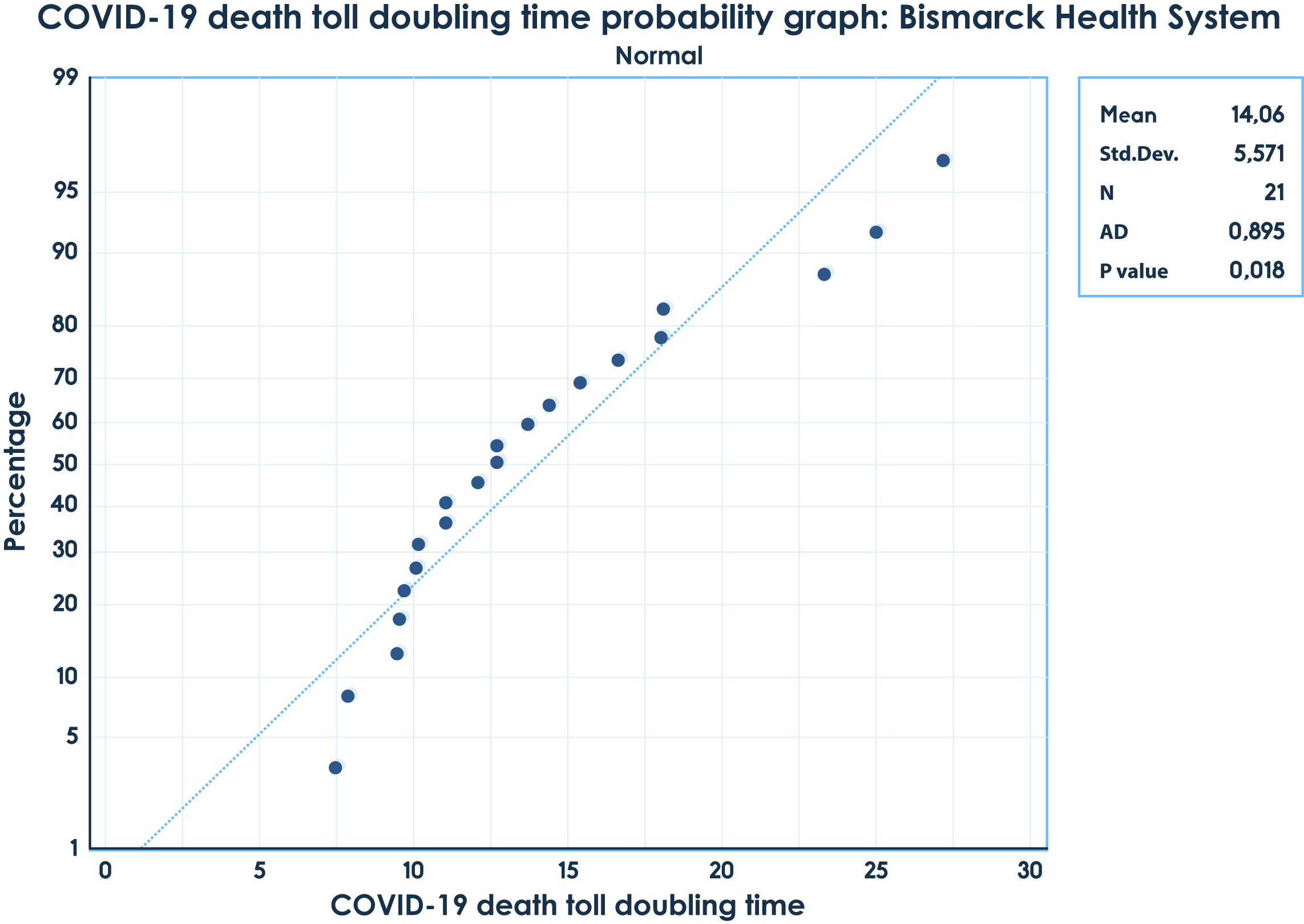

Supplement: Supplementary Figure 1 — Anderson-Darling goodness-of-fit test for general country data. [file Image_1.TIF]

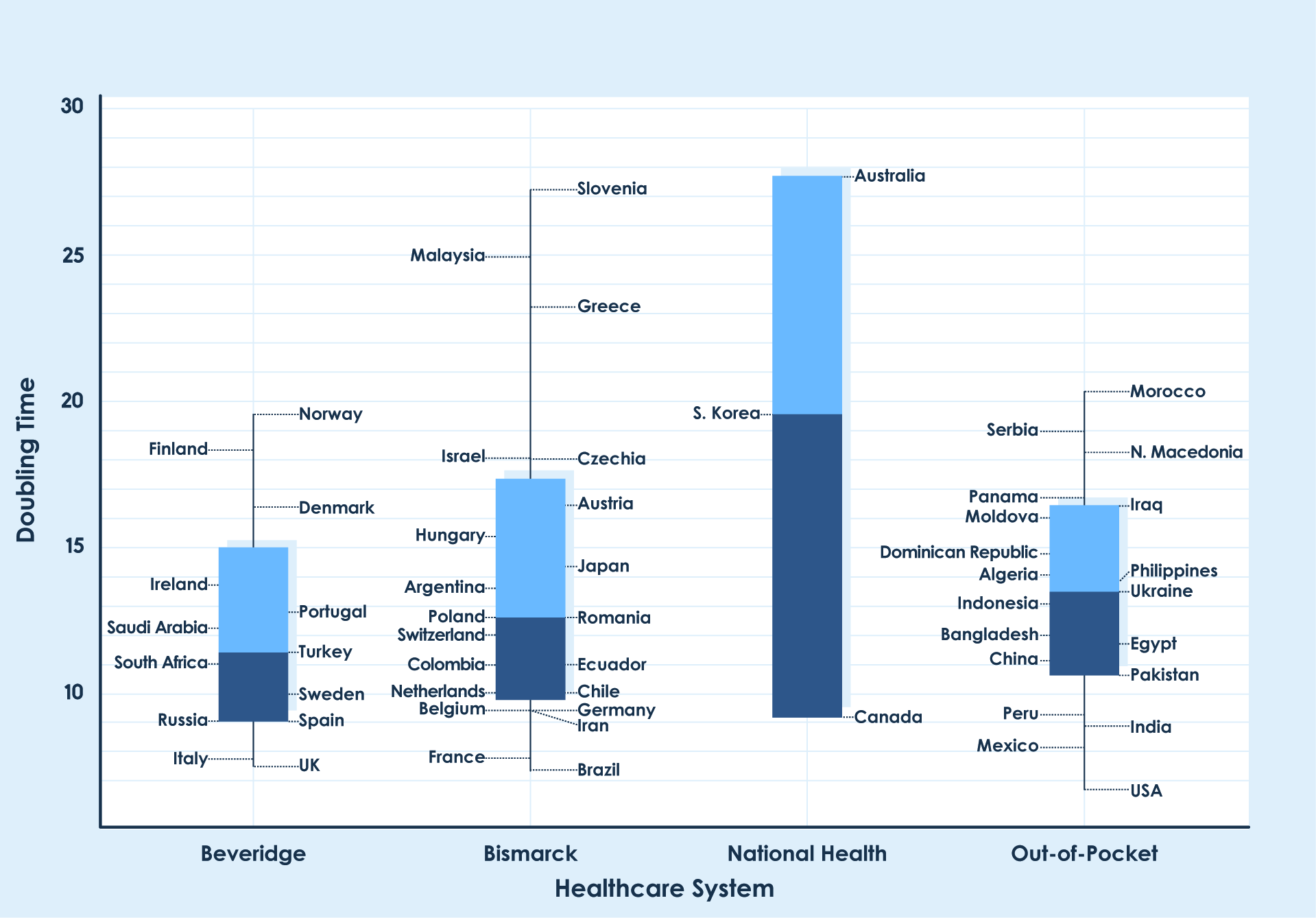

Supplement: Supplementary Figure 2 — Box Plot for doubling time of deaths with period length 90 days. [file Image_2.TIF]
